# Genetic Variant of *AMD1* Is Associated with Obesity in Urban Indian Children

**DOI:** 10.1371/journal.pone.0033162

**Published:** 2012-04-09

**Authors:** Rubina Tabassum, Alok Jaiswal, Ganesh Chauhan, Om Prakash Dwivedi, Saurabh Ghosh, Raman K. Marwaha, Nikhil Tandon, Dwaipayan Bharadwaj

**Affiliations:** 1 Genomics and Molecular Medicine Unit, CSIR-Institute of Genomics and Integrative Biology, Delhi, India; 2 Human Genetics Unit, Indian Statistical Institute, Kolkata, India; 3 Department of Endocrinology and Thyroid Research, Institute of Nuclear Medicine and Allied Sciences, Delhi, India; 4 Department of Endocrinology, All India Institute of Medical Sciences, New Delhi, India; University of Bonn, - Institute of Experimental Hematology and Transfusion Medicine, Germany

## Abstract

**Background:**

Hyperhomocysteinemia is regarded as a risk factor for cardiovascular diseases, diabetes and obesity. Manifestation of these chronic metabolic disorders starts in early life marked by increase in body mass index (BMI). We hypothesized that perturbations in homocysteine metabolism in early life could be a link between childhood obesity and adult metabolic disorders. Thus here we investigated association of common variants from homocysteine metabolism pathway genes with obesity in 3,168 urban Indian children.

**Methodology/Principal Findings:**

We genotyped 90 common variants from 18 genes in 1,325 children comprising of 862 normal-weight (NW) and 463 over-weight/obese (OW/OB) children in stage 1. The top signal obtained was replicated in an independent sample set of 1843 children (1,399 NW and 444 OW/OB) in stage 2. Stage 1 association analysis revealed association between seven variants and childhood obesity at *P*<0.05, but association of only rs2796749 in *AMD1* [OR = 1.41, *P* = 1.5×10^-4^] remained significant after multiple testing correction. Association of rs2796749 with childhood obesity was validated in stage 2 [OR = 1.28, *P* = 4.2×10^-3^] and meta-analysis [OR = 1.35, *P* = 1.9×10^-6^]. *AMD1* variant rs2796749 was also associated with quantitative measures of adiposity and plasma leptin levels that was also replicated and corroborated in combined analysis.

**Conclusions/Significance:**

Our study provides first evidence for the association of *AMD1* variant with obesity and plasma leptin levels in children. Further studies to confirm this association, its functional significance and mechanism of action need to be undertaken.

## Introduction

Childhood obesity is a growing public health issue worldwide [1]. Prevalence of overweight/obesity has increased from 16% in 2002 to 24% in 2006 in urban school children in Delhi, India [2]. Both genetic and environmental factors play an important role in the development of obesity. Studies investigating the genetic component of obesity have primarily focused on adult obesity and around 32 obesity susceptibility genes have been identified through large scale genome wide association studies (GWAS) [3]. The search for genetic risk factors for childhood obesity and related phenotypes are mainly limited to the replication of variants identified through genome wide association studies (GWAS) in adults [3].

Childhood obesity is one of the major determinants of many chronic diseases in adulthood such as type 2 diabetes, hypertension and cardio-vascular diseases [4]. Manifestation of these chronic metabolic disorders, which have become pandemic in India [5]–[7], starts in early life [8]. Indian subjects with impaired glucose tolerance or diabetes are shown to have typically low BMI up to the age of two years, followed by increasing BMI at the age of 12 years [8]. This suggests that molecular events leading to obesity in childhood predispose individuals to chronic metabolic disorders in later life. This makes identification of factors linking childhood obesity and adult chronic disorders very imperative.

**Figure 1:**
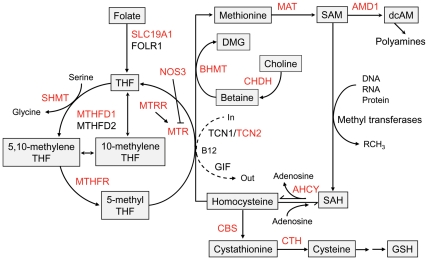
Homocysteine metabolism pathway. The genes selected for the study are marked in red fonts. Refer to Table 2 for gene names. Figure is adapted from Souto JC et al. (2005) Am J Hum Genet 76: 925–933 and Finkelstein JD (1998) Eur J Pediatr 157: S40–44.

Elevated level of homocysteine, termed as hyperhomocysteinemia, is regarded as a potential risk factor for cardiovascular diseases, diabetes, hypertension and number of other pathologies [9]–[12]. Homocysteine is a thiol containing amino acid which plays an important role in cell metabolism. The metabolic traffic of homocysteine occurs either via remethylation to methionine or through irreversible trans-sulfuration to cysteine. Homocysteine metabolism involves a series of enzymatic reactions that produce variety of metabolic intermediates which are important for cellular processes such as transmethylation, transulfuration and polyamine biosynthesis (Figure 1) [13]. Perturbations in the activities of enzymes involved in these processes such as methylene tetrahydrofolate reductase (MTHFR), methylene tetrahydrofolate dehydrogenase (MTHFD), methionine synthase reductase (MTRR), cystathionine bsynthase (CBS) may results in altered levels of homocysteine and thus metabolic disorders.

Contribution of variants in homocysteine pathway genes to susceptibility of diabetes, obesity and vascular diseases has been suggested by previous studies [14]–[16]. Genetic variants of *MTHFR*, *MTR* and *MTRR* have been shown to be associated with obesity [17]. The C677T polymorphism of *MTHFR* is reported to be associated with hyperhomocysteinemia, type 2 diabetes and related complications [18], [19]. However there is no report of association of homocysteine pathway genes with childhood obesity. Here we hypothesized that genetic variant in homocysteine metabolism pathway genes leading to perturbation in homocysteine metabolism might be a link between childhood obesity and adult metabolic disorders. To test this, we designed the present two stage case-control study to investigate association of 90 common variants from homocysteine metabolism pathway genes with obesity in 3,168 urban Indian children.

## Materials and Methods

### Ethics Statement

The study was carried out in accordance with the principles of Helsinki Declaration and was approved by Human Ethics Committee of Institute of Genomics and Integrative Biology (CSIR) and All India Institute of Medical Sciences Research Ethics Committee. Informed written consent was obtained from all the participants. Prior informed written consent was obtained from parents/guardians of the children while verbal consent from children themselves was taken.

### Study Population

A total of 3,168 children (aged 11–17 years) were recruited from school health survey from four different zones of Delhi (India) after obtaining prior informed consent from school authorities, parents/guardians and verbal consent from children themselves. For stage 1, we selected 1,325 children [862 NW and 463 OW/OB] to identify SNPs showing association with obesity. This was followed by further selection of 1843 children (1,399 NW and 444 OW/ OB) in stage 2 for replication analysis. Subjects were classified as normal-weight (NW) and over-weight/obese (OW/OB) based on age and sex specific BMI cutoff provided by Cole et al [20], that gives BMI cut offs for overweight and obesity by sex for children between 2 and 18 years corresponding to the cut off points of 25 and 30 kg/m^2^ for adults.

### Anthropometric and Clinical Measurements

Anthropometric measurements including height, weight, waist circumference (WC) and hip circumference (HC) were taken using standard methods. BMI and waist-to-hip ratio (WHR) were calculated. Plasma levels of glucose, insulin and C-peptide were measured as described earlier [21] and HOMA-IR was calculated as described by Matthews et al. [22]. Plasma levels of leptin, resistin and adiponectin were measured using commercial ELISA kits (R&D Systems, Minneapolis, MN). Anthropometric and clinical characteristics of subjects are provided in Table 1.

**Table 1:** Clinical characteristics of subjects.

Character	Stage 1		Stage 2		*P* ^†^	*P* ^‡^
	NW children	OW/OB children	NW children	OW/OB children		
N (M/F)	830 (370/464)	453 (173/279)	1399 (420/979)	444 (132/312)		
Age (years)	14.00 (12.50–15.00)	13.00 (12.00–15.00)	13.00 (12.00–14.50)	13.2 (12.0–15.0)	3.0×10^-17^	0.43
Height (m)	1.54 (1.48–1.61)	1.56 (1.50–1.62)	1.52 (1.46–1.58)	1.56 (1.51–1.62)	4.8×10^-8^	0.24
Z-Height*	-0.11±0.99	0.27±0.89	-0.11±1.00	0.35±0.93	0.93	0.18
Weight (kg)	42.30 (37.00–48.85)	64.00 (55.45–73.90)	42.00 (36.00–48.40)	63.5 (56.0–71.0)	0.18	0.73
Z-Weight*	-0.51±0.75	1.20±0.54	-0.40±0.70	1.17±0.55	4.4×10^-4^	0.35
BMI (kg/m^2^)	17.58 (15.86–19.44)	25.85 (23.97–28.97)	17.91 (16.26–19.78)	25.6 (24.0–28.0)	2.9×10^-3^	0.14
Z-BMI*	-0.56±0.76	1.27±0.49	-0.40±0.65	1.18±0.50	7.3×10^-7^	0.01
WC (m)	0.66 (0.61–0.72)	0.84 (0.77–0.90)	0.65 (0.60–0.71)	0.85 (0.78–0.90)	4.2×10^-5^	1.2×10^-3^
Z-WC*	-0.42±0.77	1.11±0.59	-0.40±0.79	1.11±0.63	1.49×10^-4^	2.7×10^-13^
HC (m)	0.81 (0.76–0.86)	0.97 (0.92–1.04)	0.80 (0.75–0.86)	0.97 (0.91–1.02)	8.3×10^-3^	2.1×10^-8^
Z-HC*	-0.42±0.76	1.23±0.63	-0.41±0.71	1.11±0.62	0.11	1.4×10^-14^
WHR	0.82 (0.78–0.87)	0.86 (0.81–0.90)	0.82 (0.78–0.86)	0.87 (0.83–0.92)	0.05	0.21
Z-WHR*	-0.22±0.89	0.41±0.93	-0.20±0.98	0.62±0.96	0.14	1.3×10^-9^
Glucose (mmol/L)	5.04 (4.72–5.33)	5.00 (4.72–5.33)	4.77 (4.44–5.11)	4.60 (4.22–4.90)	3.3×10^-23^	6.3×10^-27^
Insulin (pmol/L)	37.80 (23.40–54.60)	76.80 (52.20–108.00)	42.00 (28.80–58.80)	71.40 (47.40–111.60)	4.4×10^-5^	1.8×10^-3^
C-peptide (nmol/L)	0.46 (0.33–0.59)	0.61 (0.39–0.80)	0.48 (0.37–0.62)	0.70 (0.53–0.89)	0.86	0.19
HOMA-IR	1.41 (0.83–2.09)	2.83 (1.86–4.13)	1.44 (0.99–2.09)	2.41 (1.52–3.89)	8.1×10^-3^	1.4×10^-4^
hsCRP (mg/L)	0.25 (0.1–0.76)	1.26 (0.48–3.06)	0.34 (0.13–0.84)	1.11 (0.53–2.38)	3.0×10^-3^	0.94
Adiponectin (µg/mL)	8.45 (5.95–12.50)	4.62 (2.81–7.18)	8.61 (4.80–13.59)	7.79 (4.76–10.83)	0.07	0.18
Leptin (ng/mL)	6.68 (4.30–11.51)	17.37 (10.72–26.81)	8.14 (5.10–12.75)	19.53 (13.43–30.34)	5.4×10^-5^	3.0×10^-3^
Resistin (ng/mL)	5.59 (4.39–7.82)	5.76 (4.33–7.25)	5.30 (4.25–6.80)	5.67 (4.60–7.25)	9.7×10^-3^	7.6×10^-8^

Data are presented as median with interquartile ranges.

* Data presented are mean values with standard deviation for Z-scores of the parameters.

### Genes and SNPs Selection

We selected 16 genes which have central role in the homocysteine metabolism pathway (Figure 1) [13], [23] and 2 genes (*ACE* and *NOS3*) reported to be associated with plasma homocysteine levels [24], [25]. Common variants (MAF >0.05) of the selected genes were prioritized based on previous reports of association with metabolic disorders, tagging and linkage disequilibrium (LD) pattern information, and spacing between the SNPs to cover the gene. Finally a set of 90 SNPs were selected for genotyping in stage 1 (Table S1).

### Genotyping

Genotyping in stage 1 was performed using Illumina GoldenGate assay (Illumina Inc., San Diego, CA, USA). Stringent quality control (QC) were applied to the genotyping data that included genotype confidence score >0.25, SNP call rate >0.9, GenTrans score >0.6, cluster separation score >0.4, Hardy Weinberg Equilibrium (HWE) [*P>*0.01 in NW, OW/OB and all subjects], minor allele frequency (MAF) >0.05. After excluding 35 SNPs based on QC criteria (Table S1), the successfully genotyped SNPs had an average call rate of 98% and concordance rate >99% based on 5% duplication.

**Table 2:** Homocysteine metabolism pathway genes and association analysis of their variants with obesity in Indian children.

Gene ID	Gene name	SNP ID	Position in gene	Base change	AA change	MAF NW	MAF OW/OB	OR (95%CI)	P
MTHFR	Methylenetetrahydrofolate reductase	rs3737965	Intron 1	G/A		0.09	0.08	0.86 (0.63–1.18)	0.36
		rs9651118	Intron 2	T/C		0.28	0.25	0.84 (0.69–1.01)	0.07
		rs1801133	Exon 4	C/T	A222V	0.17	0.20	1.24 (1.01–1.52)	0.04
		rs1801131	Exon 7	A/C	E429A	0.38	0.41	1.12 (0.95–1.32)	0.19
CTH	Cystathionase	rs663465	5' flank	C/T		0.43	0.45	1.1 (0.93–1.30)	0.27
		rs663649	Intron 7	C/A		0.18	0.19	1.04 (0.84–1.29)	0.70
		rs1021737	Exon 12	G/T	S403I	0.25	0.29	1.26 (1.05–1.52)	0.01
		rs6693082	3' flank	T/G		0.25	0.30	1.26 (1.05–1.51)	0.01
MTR	5-methyltetrahydrofolate-homocysteine	rs946403	Intron 13	A/G		0.37	0.41	1.16 (0.98–1.38)	0.09
	methyltransferase	rs1770449	Intron 24	A/G		0.30	0.28	0.91 (0.76–1.08)	0.27
		rs1805087	Exon 26	T/C	D919G	0.31	0.30	0.95 (0.79–1.13)	0.55
		rs16834521	Exon 28	A/G	A1048A	0.31	0.34	1.15 (0.97–1.37)	0.12
		rs2229276	Exon 29	A/G	A1048A	0.31	0.34	1.16 (0.97–1.38)	0.10
		rs1050993	3' UTR	C/T		0.29	0.28	0.91 (0.76–1.09)	0.31
CHDH	Choline dehydrogenase	rs6445607	Intron 1	T/G		0.25	0.27	1.14 (0.94–1.39)	0.17
		rs2241808	Exon 4	A/G	A240A	0.41	0.43	1.09 (0.92–1.28)	0.34
MTRR	Methionine synthase reductase	rs1801394	Exon 2	G/A	I22M	0.45	0.50	1.18 (1.00–1.40)	0.05
		rs162036	Exon 7	T/C	K350R	0.14	0.16	1.14 (0.90–1.43)	0.27
		rs10380	Exon 14	G/A	H595Y	0.14	0.15	1.10 (0.87–1.39)	0.42
BHMT	Betaine-homocysteine S-methyltransferase	rs492842	Intron 1	T/C		0.43	0.40	0.87 (0.74–1.03)	0.10
		rs3733890	Exon 6	C/T	R239Q	0.28	0.27	0.95 (0.78–1.14)	0.56
		rs585800	3' UTR	A/T		0.14	0.13	0.87 (0.68–1.12)	0.27
AMD1	Adenosylmethionine decarboxylase 1	rs2796749	5' flank	C/G		0.34	0.27	0.71 (0.59–0.85)	1.5×10-4
		rs1007274	Intron 1	G/A		0.24	0.27	1.10 (0.91–1.33)	0.31
		rs7768897	Intron 4	C/T		0.25	0.20	0.77 (0.63–0.93)	7.5×10-3
NOS3	Nitric oxide synthase 3	rs1799983	Exon 7	G/T	D298E	0.17	0.19	1.14 (0.92–1.41)	0.22
		rs2566514	Exon 18	G/C	A666A	0.35	0.34	0.94 (0.80–1.12)	0.50
MAT1A	Methionine adenosyltransferase I	rs17677908	5' flank	T/C		0.26	0.29	1.16 (0.96–1.39)	0.12
		rs2282367	Intron 4	G/A		0.15	0.14	0.94 (0.75–1.19)	0.63
		rs10788546	Exon 7	C/T	V290V	0.16	0.15	0.97 (0.77–1.22)	0.79
		rs2993763	Exon 9	A/G	Y377Y	0.38	0.35	0.88 (0.74–1.04)	0.14
		rs1985908	3' UTR	T/C		0.48	0.42	0.78 (0.66–0.92)	4.0×10-3
FOLH1	Folate hydrolase 1	rs202719	Intron 11	T/C		0.11	0.13	1.24 (0.97–1.59)	0.086
		rs6485965	3' flank	G/A		0.38	0.37	0.96 (0.80–1.15)	0.67
MTHFD1	Methylenetetrahydrofolate dehydrogenase 1	rs8006686	Intron 2	T/C		0.17	0.17	0.99 (0.79–1.23)	0.90
		rs1950902	Exon 6	G/A)	K134R	0.08	0.07	1.00 (0.72–1.38)	0.99
		rs8016556	Intron 16	A/G		0.26	0.24	0.90 (0.74–1.09)	0.27
		rs2236225	Exon 20	T/C	R653Q	0.50	0.49	0.93 (0.78–1.11)	0.41
		rs2281603	Intron 26	T/C		0.21	0.20	0.95 (0.78–1.17)	0.64
MTHFD1L	Methylenetetrahydrofolate dehydrogenase 1-like	rs9397028	5' flank	G/A		0.42	0.37	0.80 (0.67–0.95)	0.01
		rs2073063	Intron 4	A/G		0.43	0.39	0.84 (0.71–1.00)	0.05
		rs1555179	Intron 15	C/T		0.24	0.23	0.95 (0.79–1.15)	0.61
		rs509474	Exon 23	G/C	S832S	0.33	0.32	0.98 (0.81–1.17)	0.78
		rs1047662	3' UTR	C/G		0.28	0.27	0.97 (0.80–1.16)	0.71
SHMT1	Serine hydroxymethyltransferase 1	rs2273028	Intron 7	C/T		0.16	0.17	1.01 (0.81–1.25)	0.94
		rs1979277	Exon 12	G/A	L474F	0.16	0.15	0.99 (0.80–1.24)	0.96
		rs12949119	3' UTR	A/T		0.36	0.35	0.97 (0.81–1.16)	0.71
ACE	Angiotensin I converting enzyme	rs4291	5' flank	A/T		0.39	0.39	1.03 (0.87–1.23)	0.71
		rs4331	Exon 16	C/T	A731A	0.41	0.43	1.13 (0.96–1.33)	0.16
		rs4362	Exon 24	C/T	F1129F	0.35	0.38	1.16 (0.97–1.39)	0.10
AHCY	Adenosylhomocysteinase	rs819146	5' UTR	A/C		0.22	0.21	0.94 (0.77–1.15)	0.56
		rs819147	Intron 2	T/C		0.23	0.21	0.89 (0.73–1.09)	0.25
		rs864702	Intron 10	C/T		0.23	0.21	0.88 (0.72–1.08)	0.22
		rs819173	3' flank	T/C		0.24	0.21	0.88 (0.72–1.08)	0.21
CBS	Cystathionine-beta-synthase	rs706208	3' UTR	T/C		0.38	0.37	0.94 (0.80–1.12)	0.51
		rs6586282	Intron 15	G/A		0.08	0.07	0.89 (0.65–1.20)	0.43
		rs2014564	Intron 12	G/A		0.47	0.46	0.98 (0.83–1.16)	0.83
		rs234706	Exon 9	C/T	Y233Y	0.23	0.24	1.05 (0.87–1.28)	0.60
SLC19A1	Solute carrier family 19 member 1	rs1051296	3' UTR	A/C		0.47	0.45	0.91 (0.77–1.07)	0.24
		rs12659	Exon 3	G/A	P114P	0.34	0.34	1.03 (0.87–1.22)	0.76
		rs1051266	Exon 2	G/A	H27R	0.38	0.38	0.99 (0.84–1.17)	0.89
TCN2	Transcobalamin II	rs5749131	5' flank	A/G		0.37	0.37	0.98 (0.83–1.16)	0.81
		rs9606756	Exon 2	T/C	I23V	0.09	0.11	1.11 (0.84–1.46)	0.47
		rs1801198	Exon 6	G/C	R259P	0.41	0.43	1.08 (0.92–1.28)	0.35
		rs9621049	Exon 7	G/A	S348F	0.08	0.09	1.18 (0.88–1.58)	0.26
		rs10418	3' UTR	G/A		0.21	0.19	0.88 (0.72–1.09)	0.24

AA: Amino acid; MAF: minor allele frequency; OR: odds ratio; CI: confidence interval. OR and P values presented were calculated using logistic regression analysis under additive model with age and sex as covariates.

In stage 2, variant showing strongest association with childhood obesity and standing multiple testing correction was validated by genotyping in an independent sample set of 1843 children (1,399 NW and 444 OW/OB) using iPLEX (Sequenom, San Deigo, CA, USA). Genotyping success rate was 94% with >99.9% consistency rate in genotype calls in 5% duplicates.

**Table 3:** Association of homocysteine metabolism pathway genes with obesity in urban Indian children.

SNP	Gene	RAF NWchildren	RAF OW/OBchildren	Obesity		BMI		
				OR (95%CI)	*P*	β	SE	*P*
**Stage 1**								
rs2796749 (C/G)	*AMD1*	0.657	0.733	1.41 (1.18–1.69)	1.5×10^-4^	0.16	0.05	4.2×10^-4^
rs7768897 (C/T)	*AMD1*	0.745	0.795	1.30 (1.07–1.59)	7.5×10^-3^	0.11	0.05	0.03
rs1985908 (T/C)	*MAT1A*	0.524	0.581	1.28 (1.09–1.51)	4.0×10^-3^	0.14	0.04	1.5×10^-3^
rs9397028 (G/A)	*MTHFD1L*	0.577	0.627	1.25 (1.05–1.49)	0.01	0.15	0.05	1.3×10^-3^
rs1021737 (G/T)	*CTH*	0.250	0.292	1.26 (1.05–1.52)	0.01	0.16	0.05	1.6×10^-3^
rs6693082 (T/G)	*CTH*	0.250	0.300	1.26 (1.05–1.51)	0.01	0.16	0.05	9.1×10^-4^
rs1801133 (C/T)	*MTHFR*	0.169	0.203	1.23 (1.01–1.52)	0.04	0.12	0.06	0.02
**Stage 2**								
rs2796749 (C/G)	*AMD1*	0.668	0.723	1.28 (1.09–1.54)	4.2×10^-3^	0.10	0.03	2.6×10^-3^
**Combined**								
rs2796749 (C/G)	*AMD1*	0.664	0.728	1.35 (1.19–1.52)	1.9×10^-6^	0.13	0.03	2.5×10^-6^
Meta-analysis*								
rs2796749 (C/G)	*AMD1*			1.35	2.7×10^-6^	0.12		6.5×10^-6^

RAF: risk allele frequency; OR: odds ratio. Odds ratio are presented with respect to risk allele that are highlighted as underlined text. β: change in Z score of BMI per increase in risk allele; SE: standard error. Combined analysis was performed by combining raw genotype data from stage 1 and stage 2. Meta-analysis was performed by combining the summary estimates (OR/β, 95%CI and S.E). No heterogeneity in two study population was observed (P value for Cochrane's Q statistic = 0.48 and Î2 heterogeneity index = 0.0). *OR/β and P value for fixed effect meta-analysis.

### Statistical Analysis

Hardy Weinberg Equilibrium (HWE) was tested using χ^2^. Pairwise LD between the SNPs and haplotype blocks were determined using Haploview 4.0. Continuous variables were inverse normal transformed and were converted to age and sex specific internal Z-scores by dividing differences of mean value of study population and individual values by standard deviation. Logistic and linear regression analyses were performed to assess associations of variants with obesity and quantitative traits respectively under additive model adjusting for age, sex and Z- BMI as appropriate. A *P* value of <7.7×10^-4^ [0.05/(65] was considered significant after Bonferroni correction. Meta-analysis was performed by combining summary estimates while association by combining raw genotyped data of two phases was also tested. β values are presented as Z-score units. For continuous traits, *P* value of <5.5×10^-5^ was considered statistically significant [0.05/(65*14)]. Haplotype association analysis adjusted for age and sex was carried out at 10,000 permutations. Statistical analyses were performed using PLINK v. 1.07 (http://pngu.mgh.harvard.edu/purcell/plink) and SPSS v. 17.0 (SPSS, Chicago, IL, USA).

Statistical power of the study was determined using Quanto software (http://hydra.usc.edu/gxe/) assuming log additive model of inheritance and 24% prevalence of overweight/obesity (1) at α = 0.05. Sample size in stage 1 and combined analysis provided 44–76% and 76–97% power respectively to detect association of variant with allele frequency of 0.20 and effect size of 1.20–1.30.

## Results

Association analysis in stage 1 suggested association of seven variants-rs2796749 (*AMD1*), rs7768897 (*AMD1*), rs1985908 (*MAT1A*), rs9397028 (*MTHFD1L*), rs1021737 (*CTH*), rs6693082 (*CTH*) and rs1801133 (*MTHFR*) with childhood obesity at *P*<0.05 (Tables 2 and 3). The association of rs2796749 in *AMD1* [OR = 1.41, *P* = 1.5×10^-4^] remained significant even after multiple testing correction. Linear regression analysis to assess the association of variants with BMI as continuous trait also revealed association of rs2796749 with BMI [β = 0.16 Z-score unit, *P* = 4.2×10^-4^] (Table 3). *AMD1* variant rs2796749 was also associated with other quantitative measures of adiposity (Table 4). With increase in per copy of risk allele G of rs2796749, significant increase in height and weight by 0.10 Z-score units (*P* = 0.02) and 0.17 (*P* = 1.3×10^-4^) respectively was observed (Table 4). Similar trend in increase in WC and HC was found with the increase in risk allele of rs2796749 [β = 0.18, *P* = 3.2×10^-5^ for WC and β = 0.18, *P* = 4.4×10^-5^ for HC]. The rs2796749 was also associated with plasma leptin level [β = 0.14, *P* = 4.2×10^-4^].

**Table 4:** Association of *AMD1* variant rs2796749 with clinical traits in urban Indian children.

	Stage 1		Stage 2		Combined		Mean		
Trait	β	*P*	β	*P*	β	*P*	CC	CG	GG
Height†	0.10	0.02	0.09	0.01	0.09	6.7×10^–4^	−0.20	0.01	0.04
Weight†	0.17	1.3×10^–4^	0.12	2.8×10^–4^	0.14	9.1×10^–8^	−0.17	−0.05	0.10
BMI†	0.16	4.2×10^–4^	0.10	2.6×10^–3^	0.13	2.5×10^–6^	−0.11	−0.06	0.10
WC†	0.18	3.2×10^–5^	0.11	2.5×10^–3^	0.14	3.2×10^–7^	−0.16	−0.06	0.10
HC†	0.18	4.4×10^–5^	0.12	3.3×10^–4^	0.16	3.7×10^–8^	−0.17	−0.05	0.12
WHR†	0.07	0.07	0.04	0.27	0.06	0.05	−0.07	−0.04	0.03
Adiponectin‡	−0.03	0.49	−0.01	0.71	−0.02	0.41	−0.08	0.04	0.00
Leptin‡	0.14	4.2×10^–4^	0.09	0.02	0.12	2.2×10^–5^	−0.01	−0.01	0.06
Resistin‡	0.01	0.82	0.02	0.60	0.01	0.74	−0.01	0.01	0.01
hsCRP‡	0.08	0.08	0.06	0.11	0.07	0.01	0.03	−0.03	0.02
Total Cholesterol‡	−0.009	0.84	0.07	0.03	0.04	0.12	0.01	−0.05	−0.003
HDL‡	−0.07	0.05	0.009	0.81	−0.03	0.35	−0.003	−0.001	−0.01
LDL‡	0.02	0.65	0.08	0.02	0.06	0.04	−0.01	−0.02	0.03
TG‡	0.06	0.10	−0.03	0.45	0.01	0.73	0.07	0.05	0.05
FPG‡	−0.03	0.47	0.002	0.94	0.07	0.01	−0.02	0.05	0.001
FPI‡	0.06	0.15	0.07	0.06	−0.01	0.67	−0.08	0.03	−0.005
C-peptide‡	0.08	0.05	0.03	0.36	0.06	0.04	−0.09	0.06	−0.01
HOMA-IR‡	0.05	0.27	0.07	0.05	0.07	0.02	−0.09	0.04	−0.01

β represents change in Z score per increase in risk allele. The mean values presented were obtained by ANCOVA analysis adjusted for appropriate covariates.

† Analysis performed after adjusting for age and sex.

‡ Analysis performed after adjusting for age, sex and BMI.

**Figure 2:**
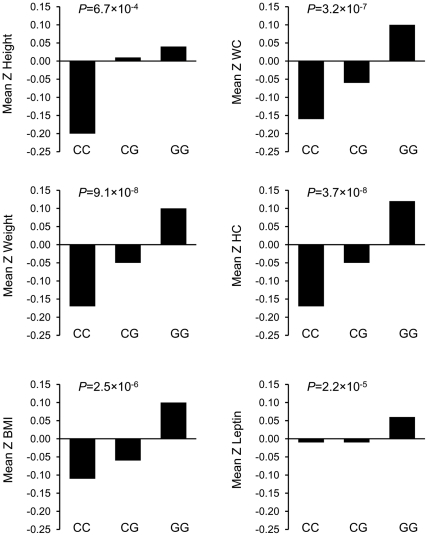
Association of rs2796749 with clinical traits in urban Indian children. Mean Z-scores adjusted for age and sex are plotted on the y-axis for the corresponding genotypes on x-axis for all the traits except leptin for which the mean Z-scores are adjusted for age, sex and Z-BMI.*P* values presented were determined by linear regression analysis for Z-score change per risk allele of the respective trait.

Because of strong association of *AMD1* variant rs2796749 with childhood obesity and measures of adiposity, we attempted to replicate the signal obtained at rs2796749 in additional sample set of 1843 children in stage 2. Association of rs2796749 with childhood obesity was validated in stage 2 [OR = 1.28, *P* = 4.2×10^–3^] and was confirmed by meta-analysis [OR = 1.35, *P* = 2.7×10^–6^] (Table 3). Similar results were obtained in combined analysis by pooling genotype data from two stages [OR = 1.35, *P* = 1.9×10^–6^]. Consistently, association of rs2796749 with BMI was also replicated [β = 0.10, *P* = 2.6×10^–3^]. The meta-analysis and combined analysis corroborated the association of rs2796749 [β = 0.12, *P* = 6.5×10^-6^ in meta-analysis and β = 0.13, *P* = 2.5× 10^–6^ in combined analysis]. Association of rs2796749 with measures of adiposity and plasma leptin levels was also replicated in stage 2 and was corroborated in combined analysis (Figure 2). No association of rs2796749 with other biochemical traits investigated could be detected (Table 4).

Stage 1 analysis also suggested association of *MAT1A* variant rs1985908 with childhood obesity [OR = 1.28, *P* = 4.0×10^–3^], BMI [β = 0.14, *P* = 1.5×10^–3^] and leptin levels [β = –0.12, *P* = 2.7×10^–3^]. *CTH* variant rs6693082 which showed nominal association with obesity, was also found to be associated with BMI [*P* = 9.1×10^–4^], WC [*P* = 4.0×10^–4^], HC [*P* = 6.3×10^-4^] and adiponectin levels [*P* = 4.5×10^–3^] (Table S2). However the associations of rs1985908 and rs6693082 with obesity and other traits did not remain significant after correcting for multiple comparisons.

Further, we investigated the association of all the SNPs genotyped in stage 1 with quantitative traits related to obesity that included measures of glucose homeostasis, lipid metabolism and adipokines. We observed suggestive association of some variants with quantitative traits (Table S2). *CTH* variant rs1021737 showed association with total cholesterol (*P* = 6.8×10^-3^]while *MAT1A* variant rs17677908 and *MTR* variant rs16834521 were found to be associated with TG [*P* = 6.8×10^-3^ and 3.56×10^-3^ respectively]. Besides *AMD1* and *MAT1A*, levels of leptin were also associated with *MTRR* variant rs1801394 [*P* = 6.8×10^-3^]. However, none of the associations remained significant after accounting for multiple tests. We also performed haplotype association analysis that suggested association of haplotypes of variants SNPs in *AMD1*, *CTH*, *MTHFR* and *MTRR* (Table S3). None of the haplotype associations remained significant after 10,000 permutations.

## Discussion

Several GWAS and large-scale meta-analysis for adult BMI have identified susceptibility loci for increased BMI, explaining only a small proportion of variation in BMI [3]. Investigation of genetic risk factors for childhood obesity has been restricted mainly to the replication of GWAS findings [3]. Though modern genotyping arrays capture most of the common variations, there remain substantial additional contributions to phenotypic variation from the variants not well captured by these arrays [26]. Investigation of variants prioritized based on their functional and positional significance provides an opportunity to identify those not detected by GWAS. Employing functional candidate analysis, we recently identified association of common variants of *IL6*, *LEPR*, and *PBEF1* with increased susceptibility to obesity and measures of adiposity in Indian children [27]. Homocysteine metabolism pathway seems to be a promising candidate pathway by virtue of its involvement in important cellular processes. Thus, here we comprehensively examined association of common variants of homocysteine metabolism pathway genes with obesity in children.

The study reveals association of *AMD1* variant rs2796749 with obesity, measures of adiposity and plasma leptin levels in urban Indian children. To the best of our knowledge, this is the first study demonstrating the association of *AMD1* variant with obesity. In our previous study of association of homocysteine metabolism pathway genes with type 2 diabetes and adult obesity [28], rs2796749 did not show association with adult obesity or BMI but was directionally consistent. Further, we investigated effect of this variant on adult BMI using the association data from GIANT consortium GWA meta-analysis which included 249,796 individuals of European ancestry [29]. In this recent meta-analysis also, rs2796749 showed consistency in direction of association with BMI, though could not reach statistical significance (*P* = 0.19). This suggests that *AMD1* variant rs2796749 might have influence on adult BMI also, but with smaller effect size.

*AMD1* codes for an important regulatory enzyme adenosyl-methionine decarboxylase 1 (AdoMetDC) which catalyzes the decarboxylation of S-adenosyl methionine (SAM), a methyl-group donor in transmethylation reactions. The imbalances in decarboxylation of SAM might result in disruption of DNA methylation process leading to abnormal gene expressions [30], which is implicated in various metabolic disorders including obesity and diabetes [31]. In addition to its role in transmethylation reaction, AdoMetDC is also a regulatory enzyme in polyamine biosynthesis. SAM is committed to polyamine bio­synthesis after decarboxylation and serves as an aminopropyl donar in spermidine and spermine synthesis [32]. The polyamines are ubiquitous and are implicated in cellular growth and differentiation, regulation of adipocyte formation and glucose metabolism [33].

A recent study reported increased levels of polyamines in childhood obesity and their correlation with biomarkers of oxidative stress, inflammation and leptin [34]. The study supported the role of polyamines on growth and development on adipose tissue. On similar lines, our study demonstrated association of rs2796749 in 5? flank region of *AMD1* with obesity in Indian children. The same variant also showed association with quantitative measures of adiposity including weight, BMI, hip and waist circumferences and plasma levels of leptin. Moreover, *AMD1* mRNA is shown to increase in response to insulin via insulin response element in 5' flank region of *AMD1* [35]. The variant rs2796749 might also have role in influencing the transcription of *AMD1* in response to insulin, which needs to be investigated.

*MAT1A* codes for methionine adenosyltransferase (MAT), which catalyzes the conversion of methionine to S- adenosylmethionine (SAM). *MAT1A* variants have been shown to be associated with hypertension and stroke [36]. Our study also suggested association of *MAT1A* variant rs1985908 with childhood obesity. *MAT1A* variants also showed association with the plasma levels of leptin, adiponectin and triglyerides. But these associations could not stand multiple testing corrections, hence further replication in larger sample set and in other ethnic groups are required to substantiate the observations in the present study.

Although the present study design limits the potential to identify the causal relationships, based on previous studies, we can speculate that *AMD1* variant might influence the susceptibility to obesity by modulating either the polyamines metabolism or DNA methylation. Dietary habits can affect these processes as nutrients such as vitamin B12 and folate are required as important cofactors in these enzymatic reactions (Figure 1). The plasma levels of folate and vitamin B12 as well as the dietary intake of these are inversely correlated with the plasma homocysteine levels [37]. Moreover, individuals consuming only vegetarian diet tend to have low vitamin B12 and elevated homocysteine levels [38], [39]. Majority of Indian population adheres to a vegetarian diet that predisposes them to vitamin B12 deficiency. Thus diet which can influence the availability of vitamins may modulate the impact of genetic variants on obesity phenotypes. Previous studies including investigation in Indian populations suggested the possibility of interaction between diet and genetic variants of homocysteine pathway genes in influencing the homocysteine levels [40], however no study provided strong evidence for this interaction and its impact on impact on phenotypes. Unfortunately, lack of data on the levels of folate and vitamin B12 and the dietary habits of our study subjects limited further investigation of the interaction of these factors with the genetic variants and their combined effect on obesity phenotypes.

In case-control association studies, limited statistical power and population stratification can lead to spurious association results. Though the sample size of our study is considerable, there is a likelihood of false negative observations for variants with small effect sizes, as the present study is sufficiently powered to capture only large effects (OR>1.3) of the common variants with frequencies more than 0.20. Further, to minimize the effect of population stratification, we have collected samples from a small geographical region that forms a homogenous cluster as reported by the Indian Genome Variation Consortium [41]. Moreover, the multidimensional scaling (MDS) analysis using the genotype data for 595 unlinked markers (r^2^<0.20) for stage 1 samples shows that the study population belongs to one cluster (Figure S1).

In conclusion, we demonstrate here, for the first time, association of *AMD1* variant with susceptibility to obesity and measures of adiposity in Indian children. Further studies to confirm the association of *AMD1* variant with childhood, its functional significance and molecular mechanism need to be undertaken. Moreover, our study provides a lead for future investigations towards understanding the genetic predisposition of obesity in childhood and exploring therapeutic options for prevention of childhood obesity.

## Supporting Information

Figure S1 **Multidimensional scaling for the study population in stage 1.** The 595 unlinked markers (r2<0.20) were used to obtain the positions on the first and second dimensions using PLINK.
(DOC)

Table S1 **SNPs selected for the study and their association with obesity in urban Indian children** AA: Amino acid; HWE: Hardy Weinberg Equillibrium; MAF: minor allele frequency; QC: quality control; OR: odds ratio; CI: confidence interval. OR and P values presented were calculated with respect to minor allele using logistic regression analysis under additive model with age and sex as covariates.
(DOC)

Table S2 **Association of analyzed SNPs with biochemical parameters in urban Indian children** β, L95 and U95 represent change in Z-score units of the parameters with per increase in risk allele with 95% confidence interval. β values are presented with respect to the minor alleles. Analysis for height, weight, BMI, WC and HC were adjusted for age and sex whereas analyses for all other parameters were adjusted for age, sex and Z-BMI.
(DOC)

Table S3 **Association of haplotypes with obesity in urban Indian children** OR: odds ratio; NW: normal-weight; OW/OB: overweight and obese.
(DOC)
