# Supplementary material for: Genetic Variant of AMD1 Is Associated with Obesity in Urban Indian Children
Source: PLoS One. 2012 Apr 9;7(4):e33162. doi: 10.1371/journal.pone.0033162 (PMC3322123; doi:10.1371/journal.pone.0033162)
Supplement: Figure S1 — Multidimensional scaling for the study population in stage 1. The 595 unlinked markers (r2<0.20) were used to obtain the positions on the first and second dimensions using PLINK. (DOC) [file pone.0033162.s001.doc]

**Figure S1:** **Multidimensional scaling (MDS) for the study population in stage 1.**


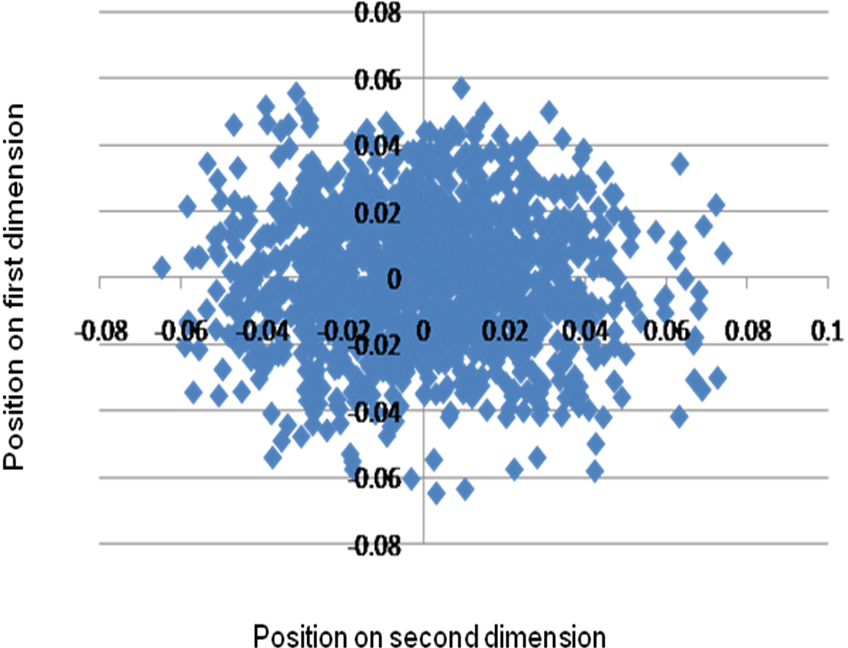


The 595 unlinked markers (r^2^ < 0.20) were used to obtain the positions on the first and second dimensions using PLINK.
