# Supplementary material for: Genetic Variant of AMD1 Is Associated with Obesity in Urban Indian Children
Source: PLoS One. 2012 Apr 9;7(4):e33162. doi: 10.1371/journal.pone.0033162 (PMC3322123; doi:10.1371/journal.pone.0033162)
Supplement: Table S1 — SNPs selected for the study and their association with obesity in urban Indian children AA: Amino acid; HWE: Hardy Weinberg Equillibrium; MAF: minor allele frequency; QC: quality control; OR: odds ratio; CI: confidence interval. OR and P values presented were calculated with respect to minor allele using logistic regression analysis under additive model with age and sex as covariates. (DOC) [file pone.0033162.s002.doc]

**Table S1:** SNPs selected for the study and their association with obesity in urban Indian children

| **S. No.** | **SNP ID** | **Chr position** | **Position in gene** | **Base change** | **AA change** | **P HWE Cases** | **P HWE Controls** | **Genotype Cases** | **Genotype Controls** | **MAF Cases** | **MAF Control** | **OR (95%CI)** | ***P*** |
| --- | --- | --- | --- | --- | --- | --- | --- | --- | --- | --- | --- | --- | --- |
| ***MTHFR* (1p36.3)** | | | | | | | | | | | | | |
| 1 | rs3737965 | 11789038 | Intron 1 | G/A |  | 0.50 | 0.11 | 1/67/373 | 2/129/611 | 0.08 | 0.09 | 0.86 (0.63-1.18) | 0.36 |
| 2 | rs9651118 | 11784801 | Intron 2 | T/C |  | 0.53 | 0.44 | 25/176/254 | 61/346/423 | 0.25 | 0.28 | 0.84 (0.69-1.01) | 0.07 |
| 3 | rs1801133 | 11778965 | Exon 4 | C/T | A222V | 0.66 | 0.08 | 20/144/290 | 31/218/581 | 0.20 | 0.17 | 1.24 (1.01-1.52) | 0.04 |
| 4 | rs1801131 | 11777063 | Exon 7 | A/C | E429A | 0.627 | 0.09 | 78/214/163 | 124/345/309 | 0.41 | 0.38 | 1.12 (0.95-1.32) | 0.19 |
| 5 | rs2274976 | 11773514 | Exon 11 | A/G | R594Q | **Illumina QC failed SNP** | | | | | | | |
| ***CTH* (1q31.1)** | | | | | | | | | | | | | |
| 6 | rs663465 | 70649346 | 5' flank | C/T |  | 1.00 | 0.67 | 93/226/135 | 151/413/265 | 0.45 | 0.43 | 1.10 (0.93-1.30) | 0.27 |
| 7 | rs672203 | 70659687 | Intron 3 | T/C |  | 0.32 | 5.5×10^-3^ | **HWE deviated SNP** | | | | | |
| 8 | rs663649 | 70669771 | Intron 7 | C/A |  | 0.88 | 0.64 | 16/136/301 | 29/240/560 | 0.19 | 0.18 | 1.04 (0.84-1.29) | 0.70 |
| 9 | rs1021737 | 70677388 | Exon 12 | G/T | S403I | 0.11 | 0.23 | 31/202/219 | 58/296/471 | 0.29 | 0.25 | 1.26 (1.05-1.52) | 0.01 |
| 10 | rs6693082 | 70678262 | 3' flank | T/G |  | 0.26 | 0.04 | 34/196/215 | 63/284/465 | 0.30 | 0.25 | 1.26 (1.05-1.51) | 0.01 |
| ***MTR* (1q43)** | | | | | | | | | | | | | |
| 11 | rs16834388 | 235023746 | 5' flank | G/T |  | **Illumina QC failed SNP** | | | | | | | |
| 12 | rs946403 | 235062740 | Intron 13 | A/G |  | 0.92 | 0.06 | 75/217/161 | 102/412/312 | 0.41 | 0.37 | 1.16 (0.98-1.38) | 0.09 |
| 13 | rs1770449 | 235104784 | Intron 24 | A/G |  | 0.42 | 0.51 | 39/176/239 | 77/337/414 | 0.28 | 0.30 | 0.91 (0.76-1.08) | 0.27 |
| 14 | rs1805087 | 235115123 | Exon 26 | T/C | D919G | 0.22 | 0.94 | 35/203/217 | 81/359/388 | 0.30 | 0.31 | 0.95 (0.79-1.13) | 0.55 |
| 15 | rs16834521 | 235121192 | Exon 28 | A/G | A1048A | 0.53 | 0.37 | 56/197/199 | 74/366/386 | 0.34 | 0.31 | 1.15 (0.97-1.37) | 0.12 |
| 16 | rs2229276 | 235121192 | Exon 29 | A/G | A1048A | 0.53 | 0.37 | 56/197/199 | 74/366/386 | 0.34 | 0.31 | 1.15 (0.97-1.37) | 0.12 |
|  | rs1050993 | 235128928 | 3' UTR | C/T |  | 0.29 | 0.51 | 40/173/240 | 76/337/416 | 0.28 | 0.29 | 0.91 (0.76-1.09) | 0.31 |
| ***CHDH* (3p21.1)** | | | | | | | | | | | | | |
| 17 | rs6445607 | 53852189 | Intron 1 | T/G |  | 0.33 | 0.57 | 27/182/232 | 45/306/456 | 0.27 | 0.25 | 1.14 (0.94-1.39) | 0.17 |
| 18 | rs12676 | 53832843 | Exon 3 | G/T | L78R | **Illumina QC failed SNP** | | | | | | | |
| 19 | rs2241808 | 53831693 | Exon 4 | A/G | A240A | 0.57 | 0.72 | 79/227/145 | 134/404/287 | 0.43 | 0.41 | 1.09 (0.92-1.28) | 0.34 |
| 20 | rs4563403 | 53825854 | 3' UTR | G/A |  | 0.57 | 6.1×10^-3^ | **HWE deviated SNP** | | | | | |
| ***MTRR* (5p15.31)** | | | | | | | | | | | | | |
| 21 | rs1801394 | 7923973 | Exon 2 | G/A | I22M | 0.51 | 1 | 106/231/110 | 169/407/244 | 0.50 | 0.45 | 1.18 (1.00-1.40) | 0.05 |
| 22 | rs1532268 | 7931179 | Exon 5 | A/G | S175L | **Illumina QC failed SNP** | | | | | | | |
| 23 | rs162036 | 7938959 | Exon 7 | T/C | K350R | 1 | 1 | 11/119/321 | 16/198/605 | 0.16 | 0.14 | 1.14 (0.90-1.43) | 0.27 |
| 24 | rs2287780 | 7942304 | Exon 9 | C/T | R415C | 1 | 1 | 0/26/429 | 0/55/776 | 0.03 | 0.03 | **MAF <0.05** | |
| 25 | rs16879334 | 7944506 | Exon 10 | G/C | P450R | 1 | 1 | 0/24/428 | 0/43/762 | 0.03 | 0.03 | **MAF <0.05** | |
| 26 | rs10380 | 7950191 | Exon 14 | G/A | H595Y | 0.58 | 0.77 | 8/118/329 | 16/193/619 | 0.15 | 0.14 | 1.10 (0.87-1.39) | 0.42 |
| 27 | rs8659 | 7953833 | 3' UTR | A/T |  | **Illumina QC failed SNP** | | | | | | | |
| ***BHMT* (5q13.1)** | | | | | | | | | | | | | |
| 28 | rs492842 | 78445743 | Intron 1 | T/C |  | 0.92 | 0.62 | 72/220/161 | 160/400/270 | 0.40 | 0.43 | 0.87 (0.74-1.03) | 0.10 |
| 29 | rs3733890 | 78457715 | Exon 6 | C/T | R239Q | 0.48 | 0.93 | 30/183/230 | 63/325/406 | 0.27 | 0.28 | 0.95 (0.78-1.14) | 0.56 |
| 30 | rs585800 | 78462964 | 3' UTR | A/T |  | 0.52 | 0.57 | 5/104/344 | 14/205/608 | 0.13 | 0.14 | 0.87 (0.68-1.12) | 0.27 |
| 31 | rs16876512 | 78443017 | 5' flank | C/T |  | 1.00 | 1.00 | 1/49/403 | 1/77/742 | 0.06 | 0.05 | **MAF <0.05** | |
| ***AMD1* (6q21)** | | | | | | | | | | | | | |
| 32 | rs2796749 | 111301396 | 5' flank | C/G |  | 0.07 | 0.49 | 40/162/251 | 102/363/362 | 0.27 | 0.34 | 0.71 (0.59-0.85) | 1.5×10^-4^ |
| 33 | rs1007274 | 111307448 | Intron 1 | G/A |  | 0.28 | 0.57 | 36/166/247 | 50/294/479 | 0.27 | 0.24 | 1.10 (0.91-1.33) | 0.31 |
| 34 | rs7768897 | 111318976 | Intron 4 | C/T |  | 0.15 | 0.58 | 24/138/292 | 56/305/457 | 0.20 | 0.25 | 0.77 (0.63-0.93) | 7.5×10^-3^ |
| ***MTHFD1L* (6q25.1)** | | | | | | | | | | | | | |
| 35 | rs9397028 | 151226920 | 5' flank | G/A |  | 0.54 | 0.19 | 58/214/170 | 130/400/250 | 0.37 | 0.42 | 0.80 (0.67-0.95) | 0.01 |
| 36 | rs2073063 | 151240764 | Intron 4 | A/G |  | 0.69 | 0.26 | 71/211/169 | 145/421/259 | 0.39 | 0.43 | 0.84 (0.71-1.00) | 0.05 |
| 37 | rs1555179 | 151313049 | Intron 15 | C/T |  | 0.18 | 0.85 | 29/150/273 | 47/290/469 | 0.23 | 0.24 | 0.95 (0.79-1.15) | 0.61 |
| 38 | rs509474 | 151378432 | Exon 23 | G/C | S832S | 0.02 | 0.87 | 35/218/199 | 86/365/374 | 0.32 | 0.33 | 0.98 (0.81-1.17) | 0.78 |
| 39 | rs1047662 | 151464528 | 3' UTR | C/G |  | 0.19 | 0.80 | 27/189/235 | 67/331/431 | 0.27 | 0.28 | 0.97 (0.80-1.16) | 0.71 |
| ***NOS3* (7q36)** | | | | | | | | | | | | | |
| 40 | rs2070744 | 150321012 | Intron 1 | C/T |  | **Illumina QC failed SNP** | | | | | | | |
| 41 | rs1549758 | 150326659 | Exon 6 | C/T | D258D | 1.00 | 1.00 | 0/0/449 | 0/0/830 | 0.00 | 0.00 | **MAF <0.05** | |
| 42 | rs1799983 | 150327044 | Exon 7 | G/T | D298E | 0.76 | 1.00 | 15/143/290 | 25/237/563 | 0.19 | 0.17 | 1.14 (0.92-1.41) | 0.22 |
| 43 | rs2566514 | 150335183 | Exon 18 | G/C | A666A | 0.11 | 0.09 | 59/184/203 | 111/346/351 | 0.34 | 0.35 | 0.94 (0.80-1.12) | 0.50 |
| ***MAT1A* (10q22)** | | | | | | | | | | | | | |
| 44 | rs17677908 | 82039583 | 5' flank | T/C |  | 0.49 | 0.10 | 34/192/226 | 62/288/455 | 0.29 | 0.26 | 1.16 (0.96-1.39) | 0.12 |
| 45 | rs2282367 | 82030326 | Intron 4 | G/A |  | 1.0 | 1.0 | 8/109/338 | 18/208/605 | 0.14 | 0.15 | 0.94 (0.75-1.19) | 0.63 |
| 46 | rs10788546 | 82024834 | Exon 7 | C/T | V290V | 0.72 | 1.00 | 9/119/327 | 20/221/588 | 0.15 | 0.16 | 0.97 (0.77-1.22) | 0.79 |
| 47 | rs2993763 | 82023574 | Exon 9 | A/G | Y377Y | 0.92 | 0.37 | 54/207/189 | 121/361/308 | 0.35 | 0.38 | 0.88 (0.74-1.04) | 0.14 |
| 48 | rs1985908 | 82022220 | 3' UTR | T/C |  | 0.28 | 0.43 | 71/225/142 | 184/381/222 | 0.42 | 0.48 | 0.78 (0.66-0.92) | 4.0×10^-3^ |
| ***FOLH1* (11p11.2)** | | | | | | | | | | | | | |
| 49 | rs202676 | 49184196 | Exon 3 | C/T | Y75H | **Illumina QC failed SNP** | | | | | | | |
| 50 | rs202680 | 49178461 | Exon 4 | A/T | A111A | **Illumina QC failed SNP** | | | | | | | |
| 51 | rs182169 | 49163891 | Exon 7 | C/T | D244D | **Illumina QC failed SNP** | | | | | | | |
| 52 | rs202719 | 49149266 | Intron 11 | T/C |  | 0.29 | 0.02 | 10/95/343 | 16/141/655 | 0.13 | 0.11 | 1.24 (0.97-1.59) | 0.086 |
| 53 | rs2299650 | 49130583 | Intron 18 | G/T |  | **Illumina QC failed SNP** | | | | | | | |
| 54 | rs6485965 | 49123306 | 3' flank | G/A |  | 0.35 | 0.70 | 52/207/166 | 103/358/290 | 0.37 | 0.38 | 0.96 (0.80-1.15) | 0.67 |
| ***MTHFD1* (14q22)** | | | | | | | | | | | | | |
| 55 | rs1076991 | 63924794 | 5' UTR | A/G |  | 0.10 | 0.02 | **HWE deviated SNP** | | | | | |
| 56 | rs8006686 | 63938424 | Intron 2 | T/C |  | 0.23 | 0.53 | 16/117/314 | 26/226/577 | 0.17 | 0.17 | 0.99 (0.79-1.23) | 0.90 |
| 57 | rs1950902 | 63952133 | Exon 6 | G/A) | K134R | 0.15 | 0.80 | 0/64/370 | 5/111/677 | 0.07 | 0.08 | 1.00 (0.72-1.38) | 0.99 |
| 58 | rs8016556 | 63973201 | Intron 16 | A/G |  | 0.90 | 0.86 | 26/162/261 | 57/315/448 | 0.24 | 0.26 | 0.90 (0.74-1.09) | 0.27 |
| 59 | rs2236225 | 63978598 | Exon 20 | T/C | R653Q | 0.03 | 0.25 | 88/232/99 | 187/407/186 | 0.49 | 0.50 | 0.93 (0.78-1.11) | 0.41 |
| 60 | rs2281603 | 63995850 | Intron 26 | T/C |  | 0.77 | 0.14 | 17/148/280 | 43/251/493 | 0.20 | 0.21 | 0.95 (0.78-1.17) | 0.64 |
| ***SHMT1* (17p11.2)** | | | | | | | | | | | | | |
| 61 | rs2273028 | 18179737 | Intron 7 | C/T |  | 0.23 | 0.07 | 16/116/316 | 29/210/580 | 0.17 | 0.16 | 1.01 (0.81-1.25) | 0.94 |
| 62 | rs1979277 | 18172821 | Exon 12 | G/A | L474F | 0.21 | 0.29 | 14/110/323 | 24/208/594 | 0.15 | 0.16 | 0.99 (0.80-1.24) | 0.96 |
| 63 | rs12949119 | 18170177 | 3' UTR | A/T |  | 0.34 | 0.16 | 47/205/179 | 87/367/307 | 0.35 | 0.36 | 0.97 (0.81-1.16) | 0.71 |
| ***ACE* (17q23.3)** | | | | | | | | | | | | | |
| 64 | rs4291 | 58907926 | 5' flank | A/T |  | 0.84 | 0.33 | 66/213/164 | 113/395/295 | 0.39 | 0.39 | 1.03 (0.87-1.23) | 0.71 |
| 65 | rs4298 | 58910932 | Exon 4 | C/T | N194N | 1.00 | 1.00 | 0/32/411 | 0/50/757 | 0.04 | 0.03 | **MAF <0.05** | |
| 66 | rs4309 | 58913655 | Exon 8 | C/T | P405P | **Illumina QC failed SNP** | | | | | | | |
| 67 | rs4331 | 58917784 | Exon 16 | C/T | A731A | 0.77 | 0.18 | 82/212/145 | 139/363/289 | 0.43 | 0.41 | 1.13 (0.96-1.33) | 0.16 |
| 68 | rs13306091 | 58920067 | Exon 18 | T/C | M828T | 1.00 | 1.00 | 0/0/451 | 0/3/825 | 0.00 | 0.00 | **MAF <0.05** | |
| 69 | rs4362 | 58927493 | Exon 24 | C/T | F1129F | 0.41 | 1.00 | 57/211/161 | 96/352/322 | 0.38 | 0.35 | 1.16 (0.97-1.39) | 0.10 |
| 70 | rs4364 | 58928394 | Exon 26 | G/T | R1286S | 1.00 | 1.00 | 0/1/452 | 0/0/825 | 0.00 | 0.00 | **MAF <0.05** | |
| ***AHCY* (20q13.1)** | | | | | | | | | | | | | |
| 71 | rs819146 | 32354861 | 5' UTR | A/C |  | 0.77 | 0.14 | 20/145/283 | 45/254/486 | 0.21 | 0.22 | 0.94 (0.77-1.15) | 0.56 |
| 72 | rs819147 | 32353365 | Intron 2 | T/C |  | 1.00 | 0.70 | 20/152/283 | 42/300/486 | 0.21 | 0.23 | 0.89 (0.73-1.09) | 0.25 |
| 73 | rs864702 | 32335211 | Intron 10 | C/T |  | 1.00 | 0.92 | 20/153/282 | 45/300/485 | 0.21 | 0.23 | 0.88 (0.72-1.08) | 0.22 |
| 74 | rs819173 | 32330223 | 3' flank | T/C |  | 1.00 | 0.92 | 20/153/278 | 45/299/478 | 0.21 | 0.24 | 0.88 (0.72-1.08) | 0.21 |
| ***CBS* (21q22.3)** | | | | | | | | | | | | | |
| 75 | rs706208 | 43346515 | 3' UTR | T/C |  | 0.76 | 0.66 | 59/214/178 | 122/381/318 | 0.37 | 0.38 | 0.94 (0.80-1.12) | 0.51 |
| 76 | rs6586282 | 43351566 | Intron 15 | G/A |  | 0.08 | 0.24 | 5/56/382 | 8/115/671 | 0.07 | 0.08 | 0.89 (0.65-1.20) | 0.43 |
| 77 | rs2014564 | 43354238 | Intron 12 | G/A |  | 0.26 | 0.78 | 103/212/137 | 179/414/229 | 0.46 | 0.47 | 0.98 (0.83-1.16) | 0.83 |
| 78 | rs234706 | 43358419 | Exon 9 | C/T | Y233Y | 0.79 | 0.05 | 26/156/249 | 51/254/459 | 0.24 | 0.23 | 1.05 (0.87-1.28) | 0.60 |
| 79 | rs397589 | 43363283 | Intron 4 | G/T |  | **Illumina QC failed SNP** | | | | | | | |
| ***SLC19A1* (21q22.3)** | | | | | | | | | | | | | |
| 80 | rs1051296 | 45759289 | 3' UTR | A/C |  | 0.92 | 0.07 | 90/223/134 | 188/369/234 | 0.45 | 0.47 | 0.91 (0.77-1.07) | 0.24 |
| 81 | rs35786590 | 45760103 | Exon 6 | C/T | A440V | 1.00 | 1.00 | 0/0/453 | 0/0/827 | 0.00 | 0.00 | **MAF <0.05** | |
| 82 | rs12659 | 45775984 | Exon 3 | G/A | P114P | 0.17 | 0.53 | 58/187/202 | 95/352/359 | 0.34 | 0.34 | 1.03 (0.87-1.22) | 0.76 |
| 83 | rs1051266 | 45782222 | Exon 2 | G/A | H27R | 0.04 | 0.55 | 73/187/182 | 123/376/314 | 0.38 | 0.38 | 0.99 (0.84-1.17) | 0.89 |
| ***TCN2* (22q12.2)** | | | | | | | | | | | | | |
| 84 | rs5749131 | 29331822 | 5' flank | A/G |  | 0.54 | 0.02 | 57/213/171 | 122/328/319 | 0.37 | 0.37 | 0.98 (0.83-1.16) | 0.81 |
| 85 | rs9606756 | 29336860 | Exon 2 | T/C | I23V | 0.31 | 0.83 | 7/79/356 | 7/128/631 | 0.11 | 0.09 | 1.11 (0.84-1.46) | 0.47 |
| 86 | rs1801198 | 29341610 | Exon 6 | G/C | R259P | 0.77 | 0.01 | 82/207/139 | 146/337/285 | 0.43 | 0.41 | 1.08 (0.92-1.28) | 0.35 |
| 87 | rs9621049 | 29343419 | Exon 7 | G/A | S348F | 0.58 | 0.47 | 5/76/374 | 3/124/701 | 0.09 | 0.08 | 1.18 (0.88-1.58) | 0.26 |
| 88 | rs4820889 | 29349044 | Exon 8 | C/T | R399Q | 0.39 | 1.00 | 1/28/423 | 1/65/764 | 0.03 | 0.04 | **MAF <0.05** | |
| 89 | rs10418 | 29352952 | 3' UTR | G/A |  | 0.22 | 0.75 | 12/150/293 | 37/268/515 | 0.19 | 0.21 | 0.88 (0.72-1.09) | 0.24 |

AA: amino acid; HWE: Hardy Weinberg Equillibrium; MAF: minor allele frequency; QC: quality control; OR: odds ratio; CI: confidence interval. OR and *P* values presented were calculated with respect to minor allele using logistic regression analysis under additive model with age and sex as covariates.
