# Supplementary material for: Genetic Variant of AMD1 Is Associated with Obesity in Urban Indian Children
Source: PLoS One. 2012 Apr 9;7(4):e33162. doi: 10.1371/journal.pone.0033162 (PMC3322123; doi:10.1371/journal.pone.0033162)
Supplement: Table S2 — Association of analyzed SNPs with biochemical parameters in urban Indian children β, L95 and U95 represent change in Z-score units of the parameters with per increase in risk allele with 95% confidence interval. β values are presented with respect to the minor alleles. Analysis for height, weight, BMI, WC and HC were adjusted for age and sex whereas analyses for all other parameters were adjusted for age, sex and Z-BMI. (DOC) [file pone.0033162.s003.doc]

**Table S2:** **Association of analyzed SNPs with biochemical parameters in urban Indian children**

| **Trait** | **SNP** | **Minor allele** | **β** | **L95** | **U95** | **P** |
| --- | --- | --- | --- | --- | --- | --- |
| Z-Height | rs1799983 | T | 0.18 | 0.08 | 0.27 | 4.6×10^-4^ |
|  | rs1985908 | C | -0.10 | -0.18 | -0.03 | 8.2×10^-3^ |
|  | rs2796749 | G | -0.10 | -0.17 | -0.02 | 0.02 |
|  | rs7768897 | T | -0.10 | -0.19 | -0.01 | 0.02 |
|  | rs9651118 | C | -0.10 | -0.18 | -0.01 | 0.03 |
|  | rs1770449 | G | -0.09 | -0.17 | 0.00 | 0.04 |
|  | rs1950902 | A | 0.15 | 0.01 | 0.30 | 0.04 |
|  | rs1050993 | T | -0.08 | -0.17 | 0.00 | 0.04 |
| Z-Weight | rs2796749 | G | -0.17 | -0.25 | -0.08 | 1.3×10^-4^ |
|  | rs1985908 | C | -0.16 | -0.25 | -0.08 | 1.8×10^-4^ |
|  | rs6693082 | G | 0.15 | 0.06 | 0.24 | 1.4×10^-3^ |
|  | rs9651118 | C | -0.15 | -0.25 | -0.06 | 1.5×10^-3^ |
|  | rs1021737 | T | 0.15 | 0.05 | 0.24 | 2.1×10^-3^ |
|  | rs9397028 | A | -0.13 | -0.22 | -0.04 | 3.7×10^-3^ |
|  | rs7768897 | T | -0.13 | -0.23 | -0.03 | 7.6×10^-3^ |
|  | rs2993763 | G | -0.11 | -0.20 | -0.03 | 8.8×10^-3^ |
|  | rs663465 | T | 0.11 | 0.03 | 0.19 | 9.9×10^-3^ |
|  | rs1799983 | T | 0.14 | 0.03 | 0.25 | 0.01 |
|  | rs1801133 | T | 0.13 | 0.03 | 0.24 | 0.01 |
|  | rs1801198 | C | 0.10 | 0.01 | 0.18 | 0.02 |
|  | rs2073063 | G | -0.10 | -0.18 | -0.01 | 0.02 |
|  | rs202719 | C | 0.15 | 0.02 | 0.27 | 0.02 |
|  | rs4362 | T | 0.10 | 0.01 | 0.19 | 0.02 |
|  | rs4331 | T | 0.09 | 0.00 | 0.17 | 0.04 |
| Z-BMI | rs2796749 | G | -0.16 | -0.25 | -0.07 | 4.2×10^-4^ |
|  | rs6693082 | G | 0.16 | 0.07 | 0.26 | 9.1×10^-4^ |
|  | rs9397028 | A | -0.15 | -0.24 | -0.06 | 1.3×10^-3^ |
|  | rs1985908 | C | -0.14 | -0.23 | -0.05 | 1.5×10^-3^ |
|  | rs2993763 | G | -0.14 | -0.23 | -0.05 | 1.6×10^-3^ |
|  | rs1021737 | T | 0.16 | 0.06 | 0.25 | 1.6×10^-3^ |
|  | rs2073063 | G | -0.11 | -0.20 | -0.03 | 0.01 |
|  | rs1801394 | A | 0.11 | 0.02 | 0.20 | 0.01 |
|  | rs9651118 | C | -0.12 | -0.22 | -0.02 | 0.01 |
|  | rs1801133 | T | 0.12 | 0.02 | 0.23 | 0.02 |
|  | rs202719 | C | 0.15 | 0.02 | 0.28 | 0.03 |
|  | rs1801198 | C | 0.10 | 0.01 | 0.19 | 0.03 |
|  | rs7768897 | T | -0.11 | -0.21 | -0.01 | 0.03 |
| Z-WC | rs2796749 | G | -0.18 | -0.26 | -0.09 | 3.0×10^-5^ |
|  | rs6693082 | G | 0.16 | 0.07 | 0.25 | 4.0×10^-4^ |
|  | rs1021737 | T | 0.16 | 0.07 | 0.25 | 5.1×10^-4^ |
|  | rs7768897 | T | -0.15 | -0.24 | -0.06 | 1.4×10^-3^ |
|  | rs1801198 | C | 0.12 | 0.04 | 0.20 | 4.1×10^-3^ |
|  | rs1555179 | T | -0.13 | -0.22 | -0.03 | 7.6×10^-3^ |
|  | rs2993763 | G | -0.11 | -0.19 | -0.02 | 0.01 |
|  | rs1985908 | C | -0.11 | -0.19 | -0.02 | 0.01 |
|  | rs9651118 | C | -0.11 | -0.21 | -0.02 | 0.01 |
|  | rs663465 | T | 0.09 | 0.01 | 0.17 | 0.03 |
|  | rs202719 | C | 0.13 | 0.01 | 0.26 | 0.04 |
|  | rs1801394 | A | 0.08 | 0.00 | 0.17 | 0.04 |
| Z-HC | rs2796749 | G | -0.19 | -0.27 | -0.10 | 4.4×10^-5^ |
|  | rs1985908 | C | -0.15 | -0.24 | -0.07 | 4.8×10^-4^ |
|  | rs6693082 | G | 0.16 | 0.07 | 0.26 | 6.3×10^-4^ |
|  | rs1021737 | T | 0.16 | 0.06 | 0.25 | 1.1×10^-3^ |
|  | rs7768897 | T | -0.15 | -0.25 | -0.05 | 2.3×10^-3^ |
|  | rs9651118 | C | -0.14 | -0.24 | -0.04 | 4.2×10^-3^ |
|  | rs9397028 | A | -0.12 | -0.21 | -0.03 | 9.8×10^-3^ |
|  | rs1801198 | C | 0.11 | 0.03 | 0.20 | 0.01 |
|  | rs2993763 | G | -0.11 | -0.20 | -0.02 | 0.01 |
|  | rs1801394 | A | 0.10 | 0.01 | 0.18 | 0.03 |
|  | rs663465 | T | 0.10 | 0.01 | 0.18 | 0.03 |
|  | rs2073063 | G | -0.09 | -0.18 | -0.01 | 0.03 |
|  | rs1799983 | T | 0.12 | 0.01 | 0.23 | 0.03 |
| Z-WHR | rs2282367 | A | -0.14 | -0.25 | -0.03 | 0.01 |
|  | rs1555179 | T | -0.12 | -0.20 | -0.03 | 0.01 |
|  | rs10788546 | T | -0.13 | -0.23 | -0.02 | 0.02 |
|  | rs3733890 | T | 0.10 | 0.01 | 0.19 | 0.02 |
| Z-Adiponectin | rs2993763 | G | 0.11 | 0.03 | 0.18 | 3.9×10^-3^ |
|  | rs6693082 | G | -0.12 | -0.20 | -0.04 | 4.5×10^-3^ |
|  | rs1021737 | T | -0.11 | -0.20 | -0.03 | 5.6×10^-3^ |
|  | rs1805087 | C | 0.10 | 0.02 | 0.18 | 0.01 |
|  | rs4362 | T | -0.10 | -0.17 | -0.02 | 0.01 |
| Z-Leptin | rs2796749 | G | -0.14 | -0.22 | -0.06 | 4.2×10^-4^ |
|  | rs1985908 | C | -0.12 | -0.19 | -0.04 | 2.7×10^-3^ |
|  | rs7768897 | T | -0.13 | -0.22 | -0.04 | 3.0×10^-3^ |
|  | rs1801394 | A | 0.10 | 0.03 | 0.18 | 6.7×10^-3^ |
|  | rs1021737 | T | 0.11 | 0.02 | 0.19 | 0.01 |
|  | rs6693082 | G | 0.10 | 0.02 | 0.19 | 0.02 |
|  | rs663465 | T | 0.08 | 0.00 | 0.15 | 0.04 |
| Z-Resistin | rs1021737 | T | -0.11 | -0.20 | -0.01 | 0.03 |
|  | rs6693082 | G | -0.10 | -0.20 | -0.01 | 0.03 |
|  | rs706208 | C | 0.09 | 0.00 | 0.18 | 0.04 |
|  | rs2241808 | G | 0.09 | 0.00 | 0.17 | 0.04 |
| Z-hsCRP | rs9651118 | C | -0.12 | -0.22 | -0.03 | 0.01 |
|  | rs2241808 | G | 0.11 | 0.02 | 0.19 | 0.01 |
|  | rs1050993 | T | -0.11 | -0.20 | -0.02 | 0.02 |
|  | rs9621049 | A | 0.18 | 0.03 | 0.33 | 0.02 |
|  | rs2566514 | C | -0.10 | -0.19 | -0.02 | 0.02 |
|  | rs1021737 | T | 0.11 | 0.02 | 0.21 | 0.02 |
|  | rs1770449 | G | -0.10 | -0.20 | -0.01 | 0.03 |
|  | rs1801133 | T | 0.12 | 0.01 | 0.22 | 0.03 |
|  | rs1979277 | A | 0.12 | 0.01 | 0.24 | 0.03 |
| Z-Cholesterol | rs1021737 | T | 0.12 | 0.03 | 0.21 | 6.8×10^-3^ |
|  | rs6693082 | G | 0.11 | 0.02 | 0.20 | 0.01 |
|  | rs16834521 | C | 0.10 | 0.02 | 0.19 | 0.02 |
|  | rs819147 | C | -0.11 | -0.20 | -0.01 | 0.03 |
|  | rs1801133 | T | 0.11 | 0.01 | 0.21 | 0.03 |
|  | rs864702 | T | -0.10 | -0.20 | -0.01 | 0.04 |
|  | rs202719 | C | 0.13 | 0.01 | 0.25 | 0.04 |
|  | rs819173 | C | -0.10 | -0.20 | 0.00 | 0.04 |
| Z-HDL | rs6445607 | G | -0.11 | -0.20 | -0.03 | 0.01 |
|  | rs2241808 | G | -0.09 | -0.16 | -0.02 | 0.02 |
|  | rs2566514 | C | 0.09 | 0.01 | 0.16 | 0.02 |
|  | rs6586282 | A | 0.15 | 0.02 | 0.28 | 0.02 |
| Z-LDL | rs492842 | C | -0.08 | -0.16 | -0.01 | 0.03 |
|  | rs819147 | C | -0.10 | -0.19 | -0.01 | 0.03 |
|  | rs1801133 | T | 0.10 | 0.01 | 0.20 | 0.03 |
|  | rs1050993 | T | -0.09 | -0.17 | -0.01 | 0.03 |
|  | rs6693082 | G | 0.09 | 0.01 | 0.18 | 0.04 |
| Z-TG | rs16834521 | C | 0.10 | 0.03 | 0.17 | 3.5×10^-3^ |
|  | rs17677908 | C | 0.10 | 0.03 | 0.17 | 6.8×10^-3^ |
|  | rs1801198 | C | 0.08 | 0.02 | 0.15 | 0.01 |
|  | rs1799983 | T | -0.10 | -0.18 | -0.01 | 0.02 |
|  | rs1985908 | C | -0.07 | -0.14 | -0.01 | 0.03 |
|  | rs9651118 | C | -0.08 | -0.15 | 0.00 | 0.04 |
| Z-FPG | rs10380 | A | -0.15 | -0.26 | -0.04 | 8.3×10^-3^ |
|  | rs16834521 | C | 0.10 | 0.02 | 0.18 | 0.02 |
|  | rs162036 | C | -0.13 | -0.24 | -0.02 | 0.02 |
| Z-C-peptide | rs162036 | C | -0.12 | -0.24 | -0.01 | 0.03 |

β, L95 and U95 represent change in Z-score units of the parameters with per increase in risk allele with 95% confidence interval. β values are presented with respect to the minor alleles.

Analysis for height, weight, BMI, WC and HC were adjusted for age and sex whereas analyses for all other parameters were adjusted for age, sex and Z-BMI.
