# Supplementary material for: Genetic Variant of AMD1 Is Associated with Obesity in Urban Indian Children
Source: PLoS One. 2012 Apr 9;7(4):e33162. doi: 10.1371/journal.pone.0033162 (PMC3322123; doi:10.1371/journal.pone.0033162)
Supplement: Table S3 — Association of haplotypes with obesity in urban Indian children OR: odds ratio; NW: normal-weight; OW/OB: overweight and obese. (DOC) [file pone.0033162.s004.doc]

**Table S3:** **Association of haplotypes with obesity in urban Indian children**

| **Gene** | **SNPs** | **Haplotypes** | **Frequency**  **NW** | **Frequency**  **OW/OB** | **OR** | ***P*** |
| --- | --- | --- | --- | --- | --- | --- |
| *AMD1* | rs2796749  rs1007274  rs7768897 | GGT  CGC  GGC | 0.26  0.42  0.09 | 0.21  0.47  0.06 | 0.76  1.26  0.67 | 6.2×10^-3^  6.2×10^-3^  0.02 |
| *CTH* | rs663649  rs1021737  rs6693082 | CGT  CTG | 0.57  0.25 | 0.52  0.29 | 0.81  1.25 | 0.02  0.02 |
| *MTHFR* | rs1801131  rs1801133 | AC | 0.45 | 0.39 | 0.80 | 9.5×10^-3^ |
| *MTHFR* | rs9651118  rs3737965 | TG | 0.63 | 0.67 | 1.21 | 0.04 |
| *MTRR* | rs1801394  rs162036  rs10380 | GTG | 0.55 | 0.51 | 0.84 | 0.04 |

OR: odds ratio; NW: normal-weight; OW/OB: overweight and obese
